# Comparison of recent updates in genetics, immunology, biomarkers, and neuroimaging of primary‐progressive and relapsing‐remitting multiple sclerosis and the role of ocrelizumab in the management of their refractory cases

**DOI:** 10.1002/hsr2.1422

**Published:** 2023-07-12

**Authors:** Priyadarshi Prajjwal, Mohammed Dheyaa Marsool Marsool, Shahnaz Asharaf, Pugazhendi Inban, Srikanth Gadam, Rukesh Yadav, Neel Vora, Varsha Nandwana, Ali Dheyaa Marsool Marsool, Omniat Amir

**Affiliations:** ^1^ Department of Neurology Bharati Vidyapeeth University Medical College Pune Pune India; ^2^ Internal Medicine Al‐Kindy College of Medicine University of Baghdad Baghdad Iraq; ^3^ Internal Medicine, Travancore Medical College Kollam India; ^4^ Internal Medicine, Government Medical College Chennai India; ^5^ Internal Medicine, Mayo Clinic Rochester New York USA; ^6^ Internal Medicine, Maharajgunj Medical Campus Tribhuvan University Kathmandu Nepal; ^7^ Internal Medicine, B.J. Medical College Ahmedabad India; ^8^ Department of Neurology Virginia Tech Carilion School of Medicine Roanoke Virginia USA; ^9^ Internal Medicine, Al Manhal Academy Khartoum Sudan

**Keywords:** disease‐modifying therapy, multiple sclerosis, ocrelizumab, primary‐progressive multiple sclerosis, relapsing‐remitting multiple sclerosis

## Abstract

**Background:**

Primary‐progressive multiple sclerosis (PPMS) and relapsing‐remitting multiple sclerosis (RRMS) are two frequent multiple sclerosis (MS) subtypes that involve 10%–15% of patients. PPMS progresses slowly and is diagnosed later in life. Both subtypes are influenced by genetic and environmental factors such as smoking, obesity, and vitamin D insufficiency. Although there is no cure, ocrelizumab can reduce symptoms and delay disease development. RRMS is an autoimmune disease that causes inflammation, demyelination, and disability. Early detection, therapy, and lifestyle changes are critical. This study delves into genetics, immunology, biomarkers, neuroimaging, and the usefulness of ocrelizumab in the treatment of refractory patients of PPMS.

**Method:**

In search of published literature providing up‐to‐date information on PPMS and RRMS, this review conducted numerous searches in databases such as PubMed, Google Scholar, MEDLINE, and Scopus. We looked into genetics, immunology, biomarkers, current breakthroughs in neuroimaging, and the role of ocrelizumab in refractory cases.

**Results:**

Our comprehensive analysis found considerable advances in genetics, immunology, biomarkers, neuroimaging, and the efficacy of ocrelizumab in the treatment of refractory patients.

**Conclusion:**

Early detection, timely intervention, and the adoption of lifestyle modifications play pivotal roles in enhancing treatment outcomes. Notably, ocrelizumab has demonstrated potential in symptom control and mitigating the rate of disease advancement, further underscoring its clinical significance in the management of MS.

## 1 INTRODUCTION

The chronic inflammatory disease of the central nervous system (CNS) known as multiple sclerosis (MS) causes a progressive loss of sensory, motor, cognitive, and visual abilities.^1^, ^2^, ^3^, ^4^, ^5^ It is most prevalent in young females.^3^, ^4^ Immune‐mediated inflammation, demyelination, and axonal destruction are the pathological outcomes of MS.^5^, ^6^ A wide variety of comorbidities can affect patients diagnosed with this disorder, and a thorough awareness of these comorbidities is essential to provide complete patient treatment.^2^ Venous drainage problems, endothelial cell abnormalities, and arterial cerebral hypoperfusion are only a few of the vascular features of MS that have been studied, and the venous part of the illness is still the least investigated.^4^

While MS has multiple subtypes, relapsing‐remitting multiple sclerosis (RRMS) is the most common subtype of MS,^5^, ^6^, ^7^, ^8^ characterized by periods of partial or total recovery following instances of abrupt neurological symptoms.

Ocrelizumab, a monoclonal antibody, has been granted approval for the treatment of both RRMS and primary‐progressive multiple sclerosis (PPMS) through its targeted depletion of CD20‐positive B cells. The findings indicate that Ocrelizumab exhibits efficacy in mitigating not only the annual rate of relapse, but also the T2‐lesions, and progression in patients with RRMS. The drug was found to be effective in two Phase III clinical trials (OPERA I and OPERA II), reducing the annual rate of relapse by 46% and 47% compared to interferon beta‐1a, respectively.

## 2 METHODOLOGY

A thorough literature search was carried out utilizing electronic databases such as PubMed, MEDLINE, Scopus, and Web of Science. The search phrases included “MS subtypes,” “genetic and environmental variables linked with MS onset and progression,” “disease‐modifying therapy,” and specific areas of research interest. Relevant keywords such as “MS” AND “Recent Updates” AND/OR “PPMS” AND/OR “RRMS,” and “Ocrelizumab” were used, and searches were conducted.

Studies were considered if they matched the following requirements: (1) produced studies in peer‐reviewed journals, (2) focused on current advances in PPMS and RRMS genetics, immunology, biomarkers, and neuroimaging, and (3) evaluated the usefulness of ocrelizumab in the therapy of refractory patients. Inclusion criteria were clinical trials, observational studies, reviews, and meta‐analyses. The review did not include conference abstracts, case reports, or editorials. Only articles written in English were considered.

## 3 DISCUSSION

### 3.1 PPMS

PPMS is an MS subtype that is characterized by a gradual and steady worsening of symptoms over time without the occurrence of distinct relapses or remissions. PPMS affects approximately 10%–15% of individuals with MS and tends to be diagnosed later in life, with symptoms typically appearing in individuals over the age of 40.^9^, ^10^

The underlying mechanisms of PPMS are not yet fully understood, but research suggests that it may be influenced by a confluence of genetic and environmental determinants. Recent studies have identified specific genetic variants that may contribute to the development of PPMS, including variants in genes involved in immune regulation and myelin repair.^11^ Environmental factors such as smoking, obesity, and vitamin D deficiency have also been implicated in the development and progression of PPMS.^12^, ^13^ The clinical course of PPMS can vary widely among individuals, with some experiencing a more rapid progression of disability while others have a slower progression.^14^ Common symptoms of PPMS include progressive weakness, difficulty with balance and coordination, vision problems, and bladder and bowel dysfunction.^9^ Although there is currently no treatment for PPMS, there are a number of DMTs that can help decrease the disease's development and control symptoms. These include interferon beta‐1b, glatiramer acetate, and ocrelizumab, which was approved by the FDA in 2017 for treating PPMS.^15^ Several ongoing clinical trials also investigate new treatments for PPMS, including therapies targeting specific immune cells and pathways.^16^

#### 3.1.1 Genetics

Research into the genetics of PPMS has identified several genetic variants that may play a role in disease development as well as its progression. Recent studies have identified specific genes involved in immune regulation and myelin repair as potential targets for PPMS treatment.^17^

One of the key genetic factors associated with PPMS is the human leukocyte antigen (HLA) system, which plays a role in immune regulation. Several HLA variants have been identified as risk factors for PPMS, including HLA‐DRB115 and HLA‐DRB1501:01.^18^, ^19^ These variants have also been implicated in the development of RRMS, the most common subtype of MS.

In addition to HLA variants, other genetic factors have been associated with PPMS. In the context of PPMS, a comprehensive genome‐wide association study (GWAS) was conducted to investigate the genetic underpinnings of the disease. The study revealed the presence of multiple genetic variants that are significantly associated with PPMS. Notably, the genes IL12A and CD58 were found to harbor variants that are particularly relevant to the disease. These findings provide valuable insights into the genetic basis of PPMS and may inform the development of targeted therapies for this debilitating condition.^20^ Although CD58 is extensively expressed in both immune and nonimmune cells, it has been hypothesized that changes in immune function play a part in how the disease MS develops. This is similar to the prominent inflammatory lesions that accompany Brain demyelination. Furthermore, once the illness has begun, increased CD58 expression may guard against the onset of MS as well as moderate acute bouts of inflammatory demyelination.

Recent studies have also identified genetic variants associated with myelin repair as potential targets for PPMS treatment. A study published in 2022 identified a genetic variant in the gene encoding the protein chitinase 3‐like 1 (CHI3L1) as a potential therapeutic target for PPMS.^21^ In MS, nonlymphocytic inflammation that is of low grade in addition to active neurodegeneration may be linked to CHI3L1 and therefore linked to progressive disease.

In one study, an investigation was conducted to explore the correlations between alleles of the CRYAB gene, which encodes for abcrystallin, in a cohort of 490 individuals diagnosed with MS. The cohort included 94 individuals diagnosed with PPMS and 182 healthy controls.^22^ A disease course that is primarily progressive in nature was linked to the CRYAB‐650*C. Not only that but the CRYAB gene was also associated with an onset in the elderly, a lower volume in T2‐lesion, as well as a greater loss of brain volume CRYAB650*C allele impact on the volume of brain was found in both PPMS as well as RR/secondary progressive multiple sclerosis (SPMS) patients.

#### 3.1.2 Pathogenesis

The prevalent lesions observed in PPMS are characterized by a gradual increase in size and comprise of T cells, microglia, and macrophage‐mediated demyelination.^23^ Since the very first events in MS are the participation of several CD+ subtypes, CD8+ cells are the major lymphocytes observed in lesions, which correspond with the degree of damage to the axons.^24^ In PPMS, sCD27, an intrathecal inflammatory marker released mostly by T cells, is increased.^25^ Patients with PPMS had high TFH and Th17 activation in their serum, which was associated with the pace of progression.

The Intrathecal production of IgG, B‐cells detection within the lesions of MS, infiltration of meninges, perivascular space, and parenchyma, and B‐cell‐based treatments success in PPMS are all evidence for the involvement of B cells in PPMS.^26^ B lymphocytes have been observed to be distributed throughout the meninges. However, it has been noted that the formation of tertiary lymphoid follicles occurs only in instances of severe illness with active progression.^27^ The quantity of B‐ and plasma cells and the level of axonal damage are correlated with PPMS lesions. B cells cause disease by a variety of mechanisms, including the presentation of antigens, release of cytokine, and the production of autoantibodies.^28^ The persistence of long‐lasting antibody‐generating plasma cells, despite the use of highly efficient monoclonal anti‐CD‐20 antibodies, supports their role beyond just the production of autoantibodies. In addition to their role in autoantibody production, these cells are capable of inducing a pro‐inflammatory reaction in myeloid cells through the secretion of granulocyte‐macrophage colony‐stimulating factor and the augmentation of Th1‐ and Th17‐differentiating cytokines, namely IL‐6 and IL‐12.^29^

B‐regulatory cells' complex role in MS is demonstrated by their secretion of IL‐10, IL‐35, and TGF‐β. Breg can stop the formation of Th1 and Th17 cells, balance the Th1/Th2 axis, and reduce macrophages.^28^

The preponderance of phagocytic cells, such as macrophages, is a notable characteristic of the slowly progressing lesions observed in PPMS. The cells in question are derived from monocytes present in the bloodstream and subsequently migrate into the brain in response to blood stimulation.^30^ Through reactive oxygen species, nitric oxide, and glutamate, the pro‐inflammatory M1 plays a crucial part in both demyelination and axonal injury.^30^ Macrophages that infiltrate the CNS are capable of inducing the development of experimental autoimmune encephalomyelitis in a gradual manner. This is achieved through the sustained release of tumor‐necrosis factor (TNF) by these cells.^31^ PPMS patients had higher sCD14 levels than healthy controls, although they were equivalent to RRMS patients who were experiencing relapses rather than remission. Regardless of the foregoing, the involvement of macrophages is deemed necessary for the process of remyelination, as they facilitate the removal of damaged tissue in lesions.^30^

sCD14 levels in PPMS patients were greater than in healthy controls, but they were comparable to those in RRMS patients who were going through relapses as opposed to remission and triggered just a Th1 cell response in vitro, rather than Th1 and Th2 as in RRMS, implying a role for dendritic cells in the illness transition into the progressive phase.^32^ The potential similarities between secondary‐progressive and PPMS warrant further investigation into the possible involvement of dendritic cells in the pathogenesis of PPMS.

A main mechanism causing degeneration of neurons and loss of axons in MS is microglial activation which has drawn more attention recently. Microglia were shown to be diffusely active in PPMS lesions as well as normally appearing white and gray matter.^33^ A considerable number of oxygen‐free radicals are released when active microglia in normally‐appearing white matter form microglial nodules surrounding stressed oligodendrocytes and damaged axons. Meningeal infiltration of B cells causes microglial activation in SPMS cortical gray matter, which shows a strong connection with clinical impairment scores.

#### 3.1.3 Characteristic clinical features

The clinical features of PPMS are distinct from those of RRMS, and early diagnosis and treatment are crucial to delaying disability progression and improving patient outcomes.

One of the hallmark clinical features of PPMS is the progressive accumulation of disability. Patients with PPMS experience a gradual decline in neurological function, with symptoms such as muscle weakness, spasticity, and gait disturbance.^29^ Unlike in RRMS, these symptoms worsen steadily over time without distinct relapses or remissions. PPMS patients may also experience urinary dysfunction, sexual dysfunction, cognitive impairment, and fatigue, which can significantly impact their quality of life.^34^

PPMS typically affects patients who are older at the time of onset, with an average age of onset of around 40–50 years old.^35^ Men and women are affected equally. PPMS also appears to be more common in patients of African descent.

#### 3.1.4 Characteristic differentiating biomarkers

Biomarkers have been proposed as potential tools for early diagnosis, disease monitoring, and predicting treatment response in PPMS patients.

One of the most promising biomarkers for PPMS is the neurofilament light chain (NfL), which is a protein found in neurons and released into the CSF and blood following neuroaxonal damage. In a recent study, the utility of NfL was investigated as a biomarker for PPMS. NfL levels were shown to be considerably greater in PPMS patients when compared to healthy controls since elevated NfL levels in the blood and CSF has been linked to disease activity and the development of impairment in PPMS patients.^36^ For example, a recent study published in JAMA Neurology found that higher baseline levels of NfL in the CSF were associated with greater cognitive decline and lesion accumulation over a 2‐year period in patients with PPMS.^37^ According to a study, people with PPMS who had higher blood levels of NfL had a higher chance of becoming disabled and experiencing brain atrophy during a 5‐year period.^36^ According to these results, NfL may be a helpful biomarker for tracking the development of the illness and the effectiveness of treatment in PPMS patients.

Another potential biomarker for PPMS is a glial fibrillary acidic protein (GFAP). In a recent investigation, it was discovered that CSF GFAP concentrations exhibited a marked increase in individuals diagnosed with PPMS in comparison to those who were deemed healthy controls. Furthermore, heightened GFAP levels were linked to heightened disability and hastened disease progression.^38^ The results of this study indicate that GFAP has the potential to serve as a valuable biomarker for the purpose of tracking the advancement of disease and evaluating the efficacy of treatment in individuals with PPMS.

In addition to NfL and GFAP, other potential biomarkers for PPMS include myelin basic protein (MBP), oligoclonal bands (OCBs), and microRNAs (miRNAs). Elevated levels of MBP in the CSF have been associated with PPMS disease activity along with progression.^39^ OCBs are bands of immunoglobulins found in the CSF of some MS patients, and their presence has been associated with a greater risk of developing PPMS.^39^

#### 3.1.5 Neuroimaging

MRI plays an important role in the diagnosis and monitoring of MS. In recent years, neuroimaging studies have shed light on the differences between PPMS and RRMS subtypes (Table 1).

**Table 1:** Comparison of the most prevalent neuroimaging findings in PPMS and RRMS subtypes of multiple sclerosis.

Neuroimaging finding	PPMS	RRMS
Lesion location	Predominantly in the spinal cord and brainstem, fewer lesions in the brain	Predominantly in the brain, with fewer lesions in the spinal cord
Lesion load	Lower lesion load compared to RRMS	Higher lesion load compared to PPMS
Gray matter atrophy	More prominent gray matter atrophy	Less prominent gray matter atrophy
Cortical thinning	More widespread cortical thinning	Less widespread cortical thinning
Corticospinal tract damage	More severe damage to the corticospinal tract	Less severe damage to the corticospinal tract
Deep gray matter perfusion	Reduced perfusion in deep gray matter structures	No significant differences compared to healthy controls
Clinical‐radiological paradox	Less prominent clinical‐radiological paradox	More prominent clinical‐radiological paradox
Spinal cord abnormalities	More frequent spinal cord abnormalities	Less frequent spinal cord abnormalities

Abbreviations: PPMS, primary‐progressive multiple sclerosis; RRMS, relapsing‐remitting multiple sclerosis.

**Figure 1:**
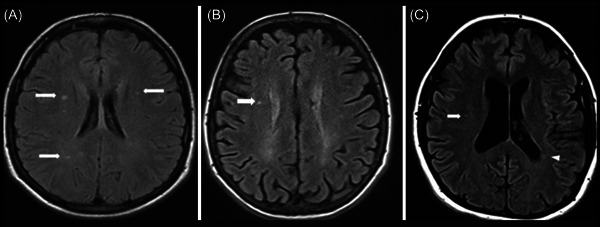
Three patients diagnosed with primary‐progressive multiple sclerosis had focal white matter changes. (A) Axial FLAIR images with a punctate lesion in the right deep white matter (white arrow); (B) axial FLAIR images with only a few small, focal white matter lesions located in the deep white matter of both the left and right brain hemispheres (white arrows); and (C) axial FLAIR images with small, focal lesions located in the right brain hemisphere's deep white matter (white arrow) and periventricular white matter, Figure from Siger.^42^

**Figure 2:**
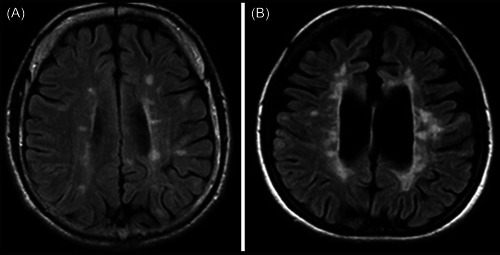
Brain MRI results in primary‐progressive multiple sclerosis (PPMS) patients are equivalent to those in relapsing‐remitting multiple sclerosis (RRMS) and secondary progressive multiple sclerosis (SPMS) patients. Brain MRI images of PPMS patients with varied FLAIR pictures, and axial plane MRI presentations. (A) The deep, juxtacortical, and periventricular white matter all had many isolated lesions. This MRI is equivalent to the standard MRI scan performed on RRMS patients; (B) An axial plane image of a FLAIR. The periventricular white matter included localized and confluent lesions, and some of the lesions in the juxtacortical regions resembled those seen in SPMS. There are indicators of brain atrophy as well, Figure from Siger.

One distinguishing feature of PPMS is the occurrence of widespread brain and spinal cord damage, which is not the case for the localized focal lesions found in RRMS. MRI techniques such as diffusion tensor imaging (DTI) and magnetization transfer imaging (MTI) can identify such diffuse, widespread damage. The DTI can be used for white matter tract integrity changes measurement, while MTI is used for the measurement of changes in the integrity of myelin.^40^, ^41^ The most common characteristic associated with MRI examination among individuals with the PPMS form of MS is a small number of focal cerebral lesions on images with T2‐weighting (Figure 1); However, PPMS patients may present with a number of focal lesions in the cerebrum in MRI, resembling those with RRMS (Figure 2). DTI investigations have revealed that individuals with PPMS had lower fractional anisotropy and higher mean diffusivity in normal‐appearing white matter, indicating extensive damage to white matter pathways.^43^, ^44^ MTI tests have also revealed extensive myelin degradation in PPMS, which may contribute to the disease's degenerative nature.^45^

In addition to these diffuse changes, neuroimaging studies have also identified specific regions of the brain that are affected in PPMS. For example, studies using functional MRI (fMRI) have shown that patients with PPMS have decreased activation in the motor cortex during a hand movement task, suggesting that there is damage to the motor pathways in these patients.^46^ Other studies have shown that the thalamus, a key relay center in the brain, is also affected in PPMS, with decreased volume and functional connectivity in this region.^47^, ^48^

### 3.2 RRMS

Approximately 85% of all cases of MS fall within the most prevalent subtype, RRMS.^49^ Relapses or exacerbations of acute neurological symptoms, also known as RRMS hallmarks, are followed by intervals of partial or total recovery, also known as remissions. In comparison to PPMS, the average age at which RRMS onset occurs is 30 years older *(*Table 2).^50^

**Table 2:** Features of relapsing‐remitting and primary‐progressive multiple sclerosis.

Characteristics	Relapsing‐remitting multiple sclerosis	Primary‐progressive multiple sclerosis
Prevalence	85%–90%	10%–15%
The mean age of onset	30 years	40 years
Female: Male ratio	2–3:1	1:1
Presenting syndrome	Optic nerve (25%), brainstem (20%), and spinal cord (45%, sensory > motor)	Spinal cord (80%, motor sensory), brainstem, and cerebellum (15%)
Gadolinium‐enhancing lesions	Common	Infrequent
CSF oligoclonal bands	Usual (~90%)	Usual (~80%)
Early spinal cord atrophy	No	Yes
Response to interferon beta	Yes (fewer relapses)	No (disability not affected)
Response to glatiramer acetate	Yes (fewer relapses)	No (disability not affected)

Abbreviation: CSF, cerebrospinal fluid.

The pathogenesis of RRMS is complex and involves genetic and environmental factors that result in an autoimmune response against the myelin sheath surrounding nerve fibers in the CNS.^51^ This autoimmune response leads to inflammation, demyelination, and axonal damage, ultimately resulting in neurological deficits and disability.

RRMS subtype has a highly unpredictable course clinically, with some patients experiencing only a few relapses and others experiencing frequent and severe relapses. Early diagnosis and treatment are crucial to prevent disability and improving long‐term outcomes in RRMS patients.

And as mentioned earlier, DMTs have been shown to reduce rates of relapse, slow progression of disability, and improve the quality of life in RRMS patients.^52^ A balanced diet, regular exercise, and stress management have all been found to have a favorable effect on the clinical course of RRMS in addition to drug therapies.

#### 3.2.1 Genes and related genetic factors

Several GWAS have identified several genetic risk factors for RRMS. These include the gene complex of HLA chromosome 6, which has been connected to a higher risk of RRMS development. Other genes that have been associated with RRMS include the interleukin 7 receptor (IL‐7R) gene, which has a role in immunological control, as well as the CD40 gene, which promotes immune activation.^53^

In addition to the above‐mentioned genes, several other factors have been implicated in the development of RRMS from a genetic point of view. These include variations in genes involved in immune regulation and inflammation, such as the IL2RA gene, the TNF‐α gene, and the IL12RB1 gene.^54^

The likelihood of having RRMS has also been linked to variations in the TNFRSF1A gene. This gene encodes for a protein involved in inflammation and immune regulation, and variants that increase its activity are more common in individuals with RRMS.^54^

Environmental factors, such as viral infections and deficiency in vitamin D, also have been implemented in the development of RRMS. For example, the Epstein‐Barr virus has been implicated as a potential trigger for RRMS, as individuals with higher levels of EBV antibodies are at an increased risk of RRMS development.^55^ Vitamin D deficiency has also been associated with an increased risk of developing RRMS, possibly due to its role in regulating immune function.^56^

#### 3.2.2 Pathogenesis

A complex interaction of genetic, environmental, and immunological variables is involved in the pathophysiology of RRMS, and it eventually results in the activation of immune cells and their subsequent attack on the myelin sheath and axons in the CNS.^57^

CD4 + T cells have been implicated in initiating and maintaining the inflammatory response in RRMS, with both Th1 and Th17 subsets playing a role.^58^ Th1 cells are involved in activating macrophages/microglia, which produce pro‐inflammatory cytokines and free radicals that contribute to the destruction of myelin and axons. Th17 cells are involved in recruiting other immune cells to the CNS and in activating B cells, which can produce antibodies that target myelin antigens.^58^

B‐cells are also involved in the pathophysiology of RRMS, as they can produce autoantibodies that target myelin antigens and contribute to the inflammatory response in the CNS. In addition, B cells can act as antigen‐presenting cells and activate T cells, further contributing to the immune response in RRMS.^59^

#### 3.2.3 Characteristic clinical features

The clinical presentation of RRMS can vary greatly between individuals, but there are some common features that are seen in most patients. The most common initial symptom of RRMS is optic neuritis.^34^ Other common initial symptoms include sensory disturbances, such as tingling or numbness in the limbs, and motor symptoms, such as weakness or spasticity.^34^

During a relapse, the symptoms may worsen or new symptoms may appear. These symptoms can affect body parts and can include weakness, numbness, tingling, visual disturbances, problems with balance and coordination, and cognitive difficulties.^60^ The severity and duration of these symptoms can vary greatly between individuals and can range from mild to severe. Patients with RRMS typically experience periods of remission between relapses, where the symptoms improve or disappear completely. However, even during periods of remission, some patients may experience residual symptoms, such as fatigue, weakness, or cognitive difficulties.^60^

#### 3.2.4 Characteristic differentiating biomarkers

In addition to NfL and GFAP, other biomarkers have been studied to differentiate between PPMS and RRMS. One such biomarker is chitinase‐3‐like protein 1 (YKL‐40), a study of which has shown that the protein is elevated in the cerebrospinal fluid of RRMS patients, but this wasn't the same for PPMS or healthy subjects. However, as noted previously, YKL‐40 was also linked to the disease progression in PPMS.^21^ Further evidence is needed to see if the YKL‐40 can be a differentiating biomarker between PPMS and RRMS.

The neurofilament heavy chain (NfH), has been found to be elevated in both RRMS and PPMS, but with higher levels in PPMS. This suggests that NfH may be a useful biomarker to differentiate between RRMS and PPMS and to monitor disease progression in PPMS.^61^

Matrix metalloproteinases (MMPs) have also been studied as potential biomarkers for MS. MMPs are enzymes that degrade extracellular matrix proteins, which play a role in the blood‐brain barrier breakdown in MS. MMP‐9 has been demonstrated in the serum of patients with RRMS to be elevated, but this is not true for patients with PPMS or healthy subjects. This suggests that MMP‐9 may be a useful biomarker to differentiate between RRMS and PPMS.^61^

Other biomarkers that have been studied to differentiate between RRMS and PPMS include osteopontin, soluble CD27, and soluble CD14. While some studies have found these biomarkers to be differentially expressed between RRMS and PPMS, more research is needed to establish their utility as diagnostic or prognostic markers.^61^

#### 3.2.5 Neuroimaging

In RRMS, MRI studies have shown that there is a link between the number of T2 lesions and the degree of clinical disability.^61^ Additionally, DTI has revealed that there is a significant correlation between the severity of impairment in cognition and the degree of white matter damage in RRMS patients.^62^

fMRI studies have also revealed differences between RRMS and PPMS patients. For example, the findings of a recent study indicated that patients exhibited a decrease in connectivity within the default mode network, a neural network that is implicated in processes related to self‐referential cognition and spontaneous thought.^63^ This reduction in connectivity was not observed in PPMS patients, suggesting that there may be differences in the pathophysiology of these two subtypes.

Optical coherence tomography studies have revealed the presence of a significant link between the degree of retinal nerve fiber layer thinning and the degree of brain atrophy in RRMS patients.^64^ This correlation was not observed in PPMS patients, indicating that there may be differences in the mechanisms underlying neurodegeneration in these two subtypes.

Magnetic resonance spectroscopy studies have also revealed differences between RRMS and PPMS patients. For example, one study found that RRMS patients had higher levels of glutamate, a neurotransmitter that has been implicated in neurotoxicity, in the normal‐appearing white matter compared to PPMS patients.^65^ This suggests that there may be differences in the inflammatory processes and neurodegenerative mechanisms between these two subtypes.

## 4 OCRELIZUMAB IN THE MANAGEMENT OF REFRACTORY CASES OF PPMS and RRMS

Recent advances in the treatment of RRMS and PPMS subtypes have been approached by the FDA‐approval of Ocrelizumab, a humanized monoclonal antibody that targets CD20‐positive B cells. The drug works by selectively depleting B cells, which are involved in the immune response and play a critical role in the pathogenesis of MS.

The efficacy of Ocrelizumab in reducing the rate of annual relapse, the number of new or enlarging lesions in T2, and the progression of disability have been demonstrated in RRMS. In the OPERA I and OPERA II clinical trials, 46% and 47% were the ability of Ocrelizumab to reduce the rate of annual relapse rate, respectively.^66^ Additionally, 95% and 95% were the percentages of reduction in new T2 lesions in both the OPERA I and OPERA II clinical trials, respectively. In the ORATORIO trial, which investigated the use of Ocrelizumab in PPMS, the drug slowed the disability progression by 24% as compared with a placebo.

**Figure 3:**
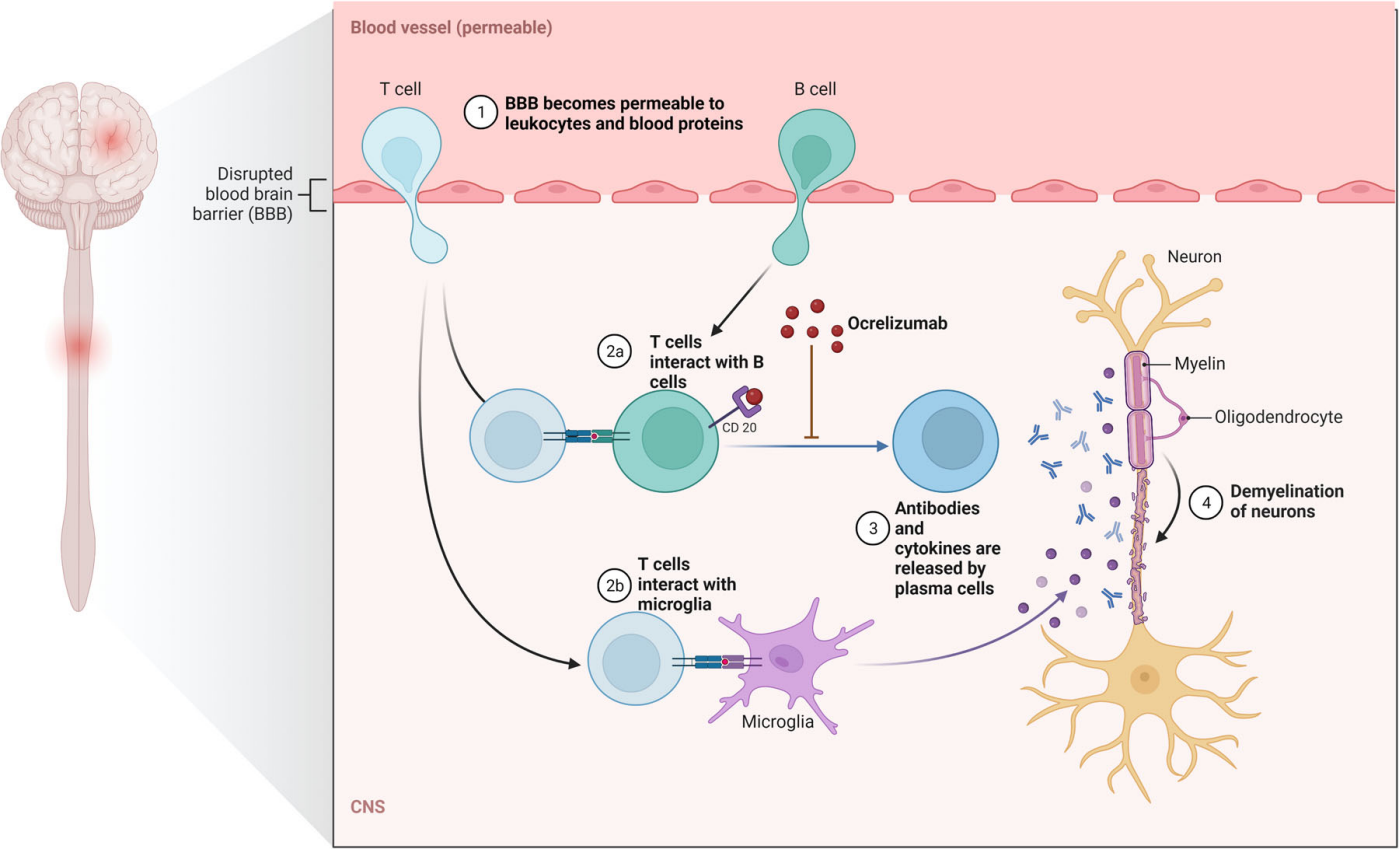
The implication of Ocrelizumab as an anti‐CD20 antibody. In multiple sclerosis (MS), the blood–brain barrier becomes permeable to blood cells and proteins, this leads to multiple interactions of T‐cells and other cells including B‐cells and microglia. The binding of Ocrelizumab to the CD‐20 receptor on B‐cells prevents them from differentiating to Plasma cells and eventually inhibit the production of antibodies implemented in the pathogenesis of MS (Original figure).

Ocrelizumab's anti‐CD20 antibody action allows it to avoid targeting plasma cells with negative CD20 on their surface while depleting circulating immature and mature B cells (Figure 3). The effector mechanisms of the anti‐CD20 antibody include complement‐dependent cytotoxicity and antibody‐dependent cellular cytotoxicity.^67^ Ocrelizumab demonstrated to result in a complete reduction in CD19+ count in B cells.^68^ A total of 72 weeks was the median duration for replenishment of B cells following the final injection of Ocrelizumab.

Ocrelizumab has been tolerated in clinical trials, with the most common adverse events being infusion reactions, infections, and infusion‐related reactions. Serious adverse events, including malignancies, have been reported but are rare. Patients treated with Ocrelizumab should be monitored for signs of infection, and immunizations should be administered before treatment initiation.

## 5 CONCLUSION

MS is a chronic inflammatory disease of the CNS that can cause damage to the myelin sheaths, leading to demyelination, axonal loss, gliosis, and neurodegeneration. PPMS and RRMS are subtypes of MS with RRMS being the most common type. Ocrelizumab, a humanized monoclonal antibody that targets CD20‐positive B cells, is approved for the treatment of both RRMS and PPMS. This drug selectively depletes B cells, which play a critical role in the pathogenesis of MS and has been shown to reduce the annualized relapse rate and the number of new or enlarging T2 lesions in RRMS.

## AUTHOR CONTRIBUTIONS

**Priyadarshi Prajjwal**: Conceptualization; methodology; validation; writing—original draft. **Mohammed Dheyaa Marsool Marsool**: Resources; validation; writing—original draft; writing—review and editing. **Shahnaz Asharaf**: Validation; writing—original draft. **Pugazhendi Inban**: Writing—original draft; writing—review and editing. **Srikanth Gadam**: Resources; supervision; validation; writing—original draft. **Rukesh Yadav**: Writing—original draft; writing—review and editing. **Neel Vora**: Visualization; writing—review and editing. **Varsha Nandwana**: Visualization; writing—original draft. **Ali Dheyaa Marsool Marsool**: Visualization; writing—original draft. **Omniat Amir**: Visualization; writing—original draft. All authors have read and approved the final version of the manuscript [Priyadarshi Prajjwal] had full access to all of the data in this study and takes complete responsibility for the integrity of the data and the accuracy of the data analysis.

## CONFLICT OF INTEREST STATEMENT

The authors declare no conflict of interest.

## TRANSPARENCY STATEMENT

The lead author Omniat Amir affirms that this manuscript is an honest, accurate, and transparent account of the study being reported; that no important aspects of the study have been omitted; and that any discrepancies from the study as planned (and if relevant, registered) have been explained.

## Data Availability

All the data used in this study are present within the study itself. No new data were created or analyzed in this study.
